# Descemet’s Membrane Detachment Following Minor Blunt Periocular Trauma: A Case Report and Review of the Literature

**DOI:** 10.7759/cureus.91930

**Published:** 2025-09-09

**Authors:** Bandar J Alharbi, Waleed Khayyat, Sara Alhilali, Lojain Albathi

**Affiliations:** 1 Ophthalmology, Makkah Health Cluster, Makkah, SAU; 2 Ophthalmology, King Khaled Eye Specialist Hospital and Research Center, Riyadh, SAU; 3 Anterior Segment, King Khaled Eye Specialist Hospital, Riyadh, SAU; 4 Ophthalmology, King Khaled Eye Specialist Hospital, Riyadh, SAU

**Keywords:** blunt ocular trauma, cornea & external eye diseases, descemet membrane detachment, literature review of disease, rare case report

## Abstract

Descemet's membrane detachment (DMD) is usually encountered postoperatively, with most cases not resolving spontaneously and requiring medical or surgical intervention. We describe a case that developed DMD immediately following blunt trauma in an aphakic eye with significant iridodonesis. The case was managed with an injection of gas tamponade that was later augmented. Eventually, the patient regained near-baseline visual acuity. DMD can occur immediately following blunt trauma with no associated endothelial abnormalities, recent intraocular surgery, keratoconus, or inflammation. Significant iridodonesis may contribute to the development of DMD in such cases.

## Introduction

Descemet's membrane detachment (DMD) is a separation of Descemet's membrane from the corneal stroma. It is usually reported in the context of a recent surgical intervention where a corneal incision was performed with an inadvertent entry into the supra-Descemet's area or a tear to the membrane [1,2]. It is a devastating complication with the potential for significant visual compromise due to corneal decompensation and scarring. In these cases, the detachment is recognized immediately during the intervention or within the early postoperative period. Very few cases have been reported with a delayed presentation of DMD months following cataract surgery [1,2]. To the best of our knowledge, after searching PubMed, Medline, Embase, and Google Scholar using the keywords Descemet Membrane Detachment, Iridodonesis, and ocular trauma, this is the first reported case of a DMD following blunt trauma in association with iridodonesis.

## Case presentation

**Figure 1:**
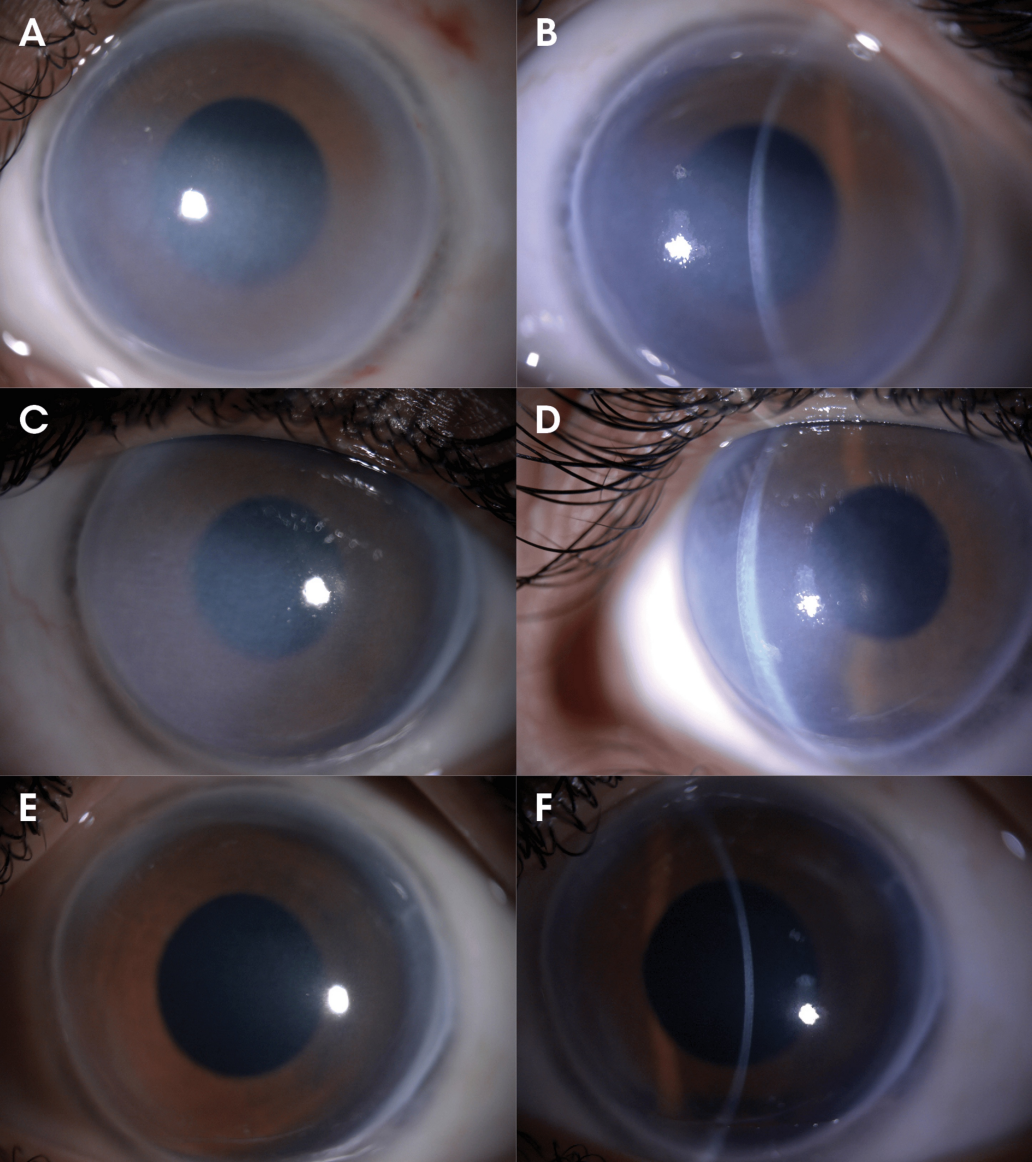
Slit lamp photos of the left eye at initial presentation showing inferior corneal edema (1A, 1B), after one month of treatment with partial improvement (1C, 1D), and at the last follow-up with total resolution (1E, 1F).

**Figure 2:**
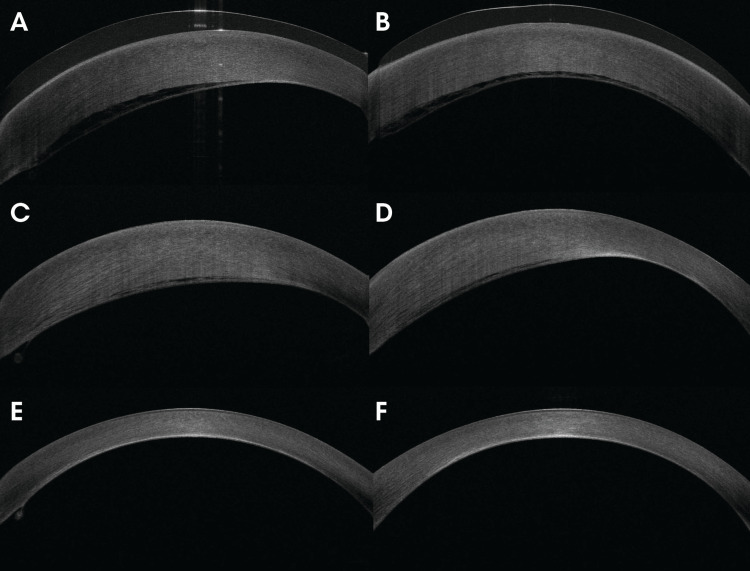
Anterior segment optical coherence tomography of the left eye at initial presentation showing Descemet's membrane detachment inferiorly with corneal edema (2A, 2B), after one month of treatment with partial improvement (2C, 2D), and at the last follow-up with total resolution (2E, 2F).

A 26-year-old female presented to our emergency room (ER) complaining of a sudden drop in vision in the left eye for five days after sustaining minor blunt trauma by the head of her child to the left side of her forehead, with no direct ocular trauma. The patient was medically fit, with no known systemic illnesses. Her past ocular history included a diagnosis of bilateral congenital cataract at the age of three years, which was successfully managed with lens aspiration, posterior capsulotomy, and anterior vitrectomy, followed by visual rehabilitation using contact lenses. At the age of 18 years, she was diagnosed with aphakic glaucoma, which was adequately controlled with topical antiglaucoma medications. She continued follow-up visits with no further complaints or documented complications. Her last documented best-corrected visual acuity (BCVA) eight months before this presentation was 20/300 for the right eye (OD) and 20/40 for the left eye (OS). Upon presenting to our ER, her visual acuity was limited to hand motion in the left eye, with an intraocular pressure of 12 mmHg in both eyes. Slit lamp examination revealed an intact closed globe with significant sectoral edema affecting the inferior hemisphere of the left cornea, impeding assessment of Descemet's membrane continuity and integrity. Both eyes were aphakic with significant iridodonesis. The remainder of the examination was unremarkable for any other sequelae of blunt ocular trauma. A slit lamp photo of the left eye is shown in Figure 1A, B. B-scan imaging confirmed a flat retina and choroid with no intraocular foreign body. Anterior segment optical coherence tomography (AS-OCT) showed a detached Descemet's membrane with no clear areas of a break or adhesions, as shown in Figure 2A, B. The endothelial cell layer could not be captured for a proper endothelial cell count assessment. However, a previous endothelial cell layer assessment done six years earlier showed a normal cell density (3531 cells/mm² OD, 3822 cells/mm² OS) with normal cell size and morphology. Ultrasound biomicroscopy was also performed, and no further abnormalities were disclosed.

Management options were discussed with the patient, and the decision was made to proceed with pneumatic descemetopexy. A bubble of 20% sulfur hexafluoride (SF6) was injected into the anterior chamber (AC) with an adequate fill of more than 80% of the anterior chamber volume, and the patient was instructed to maintain a supine position. The patient was discharged on moxifloxacin 0.5% drops four times daily for one week, cyclopentolate 1% three times daily for one week, sodium chloride 5% drops and ointment, a tapering course of prednisolone acetate 1% over four weeks starting every six hours, lubricants, a single postoperative dose of acetazolamide 250 mg, and her regular topical antiglaucoma medications (brimonidine 0.15% twice daily and dorzolamide/timolol 0.5% twice daily).

Assessment after two days showed a gas bubble filling 20% of the AC with mild corneal edema, DMD improvement, and a VA of 20/400. On the fourth day following the intervention, no further improvement was noticed except a mild decrease in corneal edema; for that reason, another AC injection of 20% SF6 was administered, and the patient was admitted to monitor her compliance with head positioning. Assessment on the following day showed a 50% gas bubble in the AC with improvement of corneal edema. On the ninth day, there was still mild corneal edema with a narrower DMD and no gas bubble left in the AC. The patient was discharged the following day on the same medications.

The findings persisted at the first outpatient visit one month after the incident with no improvement in VA (Figures 1C, 1D, 2C, 2D). Prednisolone acetate 1% was re-prescribed on a tapering dose every two weeks starting every six hours daily for eight weeks, along with sodium chloride 5% drops and antiglaucoma medications. A slit lamp photo of the cornea one month following the incident is shown in Figure 1C, D. Two months later, the examination revealed improvement of the DMD and regression of the corneal edema to only a small area inferiorly, with clearance of the visual axis resulting in a BCVA of 20/60. A gonioscopic examination was done after the corneal edema resolved, and it revealed a normal iridocorneal angle with no adhesions. A repeated attempt to measure endothelial cell count was performed but yielded only an unreliable image. At the final follow-up visit nine months after the incident, slit lamp and AS-OCT photos showed near-total regression of the edema (Figures 1E, 1F, 2E, 2F).

## Discussion

Our case demonstrates that DMD can occur after blunt trauma with no association to a recent intraocular procedure. Cataract surgery is the most common cause of diagnosed DMD cases, with an estimated incidence of 0.044-0.52% [1,2]. Associated risk factors include old age, advanced cataract, shallow AC, and endothelial dystrophy. Several cases of delayed-onset DMD after uneventful cataract surgery have been reported. In these cases, the detachment typically occurs within a few months but not exceeding a year. However, in our patient, the DMD occurred after several years, suggesting that while the lens aspiration procedure can be a risk factor, it may not be the main cause [3,4]. Previous reports of bilateral or familial cases of postoperative DMD suggest the presence of an anatomical predisposition for DMD, and this predisposition can be in the form of endothelial dysfunction or weak Descemet's adhesions to the stroma [5,6].

In a previous case report, a four-year-old child who was initially treated as a case of keratitis due to corneal opacification following an accidental fall was later discovered to actually suffer from congenital glaucoma and a traumatic DMD. It was concluded that the DMD occurred following trauma due to endothelial susceptibility caused by congenital glaucoma. Studies have shown that glaucoma and antiglaucoma medications can lead to corneal endothelial dystrophy, damage to corneal endothelial cells, and changes in Descemet's membrane [7,8]. However, our patient had normal corneal endothelial cell density and morphology.

Previous reports described DMD with a presumptive association with blunt trauma. One case was of an elderly female in whom a DMD was discovered incidentally, and upon questioning, the patient could recall only a minor unspecified blunt trauma that occurred 25 years earlier. It was not clear if the DMD occurred directly following the trauma, but a presumptive association was made by the physician [9]. Another report was of a young contact lens wearer with poor hygiene who presented with microbial keratitis surrounded by corneal edema. Upon investigation, a DMD was discovered, and the patient revealed she had sustained blunt trauma due to airbag deployment three weeks prior to presentation [10]. It is not clear how much the inflammatory process of the microbial keratitis or the exposure to alkaline material from the airbag contributed to the DMD, in addition to the mechanical trauma, in this case.

Tractional Descemet's membrane detachment has been observed following trauma caused by alkali burns, such as 50% sodium hydroxide, sodium cyanide, ammonia, and hydrogen peroxide. The proposed mechanism includes hydrogen peroxide penetrating and forming a gas bubble anterior to DM, severe cellular damage in the stroma and endothelial layer, and an inflammatory retrocorneal membrane associated with an organizing hyphema or fibrous exudates that cause traction on DM, leading to its detachment. DMD occurs after chemical burns, typically within three days to four months [11]. Gonioscopic examination of our patient revealed a normal angle with no contractile membrane.

Another case of DMD following blunt trauma was reported in an eye with a history of penetrating keratoplasty [12]. It has been described that a penetrating keratoplasty graft for keratoconus is prone to DMD, even spontaneously. The mechanism is speculated to involve thinning of the cornea, wound slippage, and recurrent ectasia [13].

Descemet's membrane injury due to bullet shockwave trauma has also been described. Bullets were discovered in the left chest and axilla of a 27-year-old male, along with a laceration on the bridge of his nose. However, no foreign bodies were found in or around the eyes. After the intraocular hemorrhage and corneal edema resolved, it became apparent that there were DM irregularities that possibly represented micro-ruptures. The authors believed that this case represented a form of sclopetaria affecting mainly the anterior segment due to the trajectory of the bullet. Another similar case of DM rupture was observed after a high-pressure water jet injury; both of these cases were treated medically [14,15].

In this report, we present a patient who developed unilateral DMD immediately following blunt trauma with no recent history of intraocular surgery, inflammation, or keratoconus. The concurrence of significant iridodonesis in our patient was unique and has not been discussed before in the context of DMD. This significant iridodonesis may have played a contributory role in the development of the DMD, as the trauma may have caused forward displacement of the iris tissue, leading to an inward mechanical displacement or break of Descemet's membrane. This is similar to the role iridodonesis is thought to play in the pathogenesis of some cases of Brown-McLean syndrome [16-18]. While we propose iridodonesis as a contributing factor, the exact mechanism remains unclear. Histological studies in aphakic eyes could clarify Descemet's membrane adhesion strength.

DMD has also been reported in newborns after forceps-assisted delivery, with the mechanism hypothesized to be due to globe compression and expansion on the opposite plane causing dehiscence of the thinner Descemet's membranes in newborns (3 microns) [19]. This mechanism is less likely to explain the DMD in our patient with a thick Descemet's membrane.

In this reported case, slit lamp examination was insufficient to detect the DMD, which was only detected through AS-OCT and was less evident on UBM. Using AS-OCT was influential throughout the follow-up visits in guiding the management decisions. This underscores the importance of using AS-OCT in cases suspected of DMD for diagnosis and monitoring, as advised by previous studies [20]. Nonetheless, even with the high resolution of AS-OCT, no break was detected in our patient. This does not necessarily exclude the presence of a break, as previous cases of small Descemet's breaks were detected only on histopathological assessment [13].

## Conclusions

DMD can occur immediately following blunt trauma with no associated endothelial abnormalities, recent intraocular surgery, keratoconus, or inflammation. The findings of this study revealed that the presence of significant iridodonesis may contribute to the development of DMD in such cases.
